# Sensing and Analyzing Partial Discharge Phenomenology in Electrical Asset Components Supplied by Distorted AC Waveform

**DOI:** 10.3390/s25216594

**Published:** 2025-10-26

**Authors:** Gian Carlo Montanari, Sukesh Babu Myneni, Zhaowen Chen, Muhammad Shafiq

**Affiliations:** Center for Advanced Power Systems (CAPS), Florida State University, Tallahassee, FL 32310, USA; gmontanari@fsu.edu (G.C.M.); zc22b@fsu.edu (Z.C.); mshafiq@fsu.edu (M.S.)

**Keywords:** non-sinusoidal AC, harmonics, notches, partial discharges, PD sensing and analytics, motors, cables, surface and internal discharges

## Abstract

Power electronic devices for AC/DC and AC/AC conversion are, nowadays, widely distributed in electrified transportation and industrial applications, which can determine significant deviation in supply voltage waveform from the AC sinusoidal and promote insulation extrinsic aging mechanisms as partial discharges (PDs). PDs are one of the most harmful processes as they are able to cause accelerated extrinsic aging of electrical insulation systems and are the cause of premature failure in electrical asset components. PD phenomenology under pulse width modulated (PWM) voltage waveforms has been dealt with in recent years, also through some IEC/IEEE standards, but less work has been performed on PD harmfulness under AC distorted waveforms containing voltage harmonics and notches. On the other hand, these voltage waveforms can often be present in electrical assets containing conventional loads and power electronics loads/drives, such as for ships or industrial installations. The purpose of this paper is to provide a contribution to this lack of knowledge, focusing on PD sensing and phenomenology. It has been shown that PD patterns can change considerably with respect to those known under sinusoidal AC when harmonic voltages and/or notches are present in the supply waveform. This can impact PD typology identification, which is based on features related to PD pattern-based physics. The adaptation of identification AI algorithms used for AC sinusoidal voltage as well as distorted AC waveforms is discussed in this paper, showing that effective identification of the type of defects generating PD, and thus of their harmfulness, can still be achieved.

## 1. Introduction

Partial discharge (PD) measurements are mostly carried out under AC sinusoidal voltage, which is, largely, the most common supply in T&D and industrial electrical assets [1,2]. Typical sensors are high-frequency current transformers (HFCTs) with bandwidths from tens of kHz to tens of MHz and capacitive couplers (typically from 80 pF to 2 nF).

Synchronization with the supply voltage zero crossing is crucial for phase-resolved PD (PRPD) pattern interpretation. Indeed, under sinusoidal AC, there is a direct correlation between the patterns and the underlying physics [3,4,5]. For example, internal discharges can occur even before supply voltage zero crossing (actually, field, since PDs are generated by the electrical field), while surface discharges generally lag. This is due to the so-called “memory effect”, that is, the electric field’s contribution of trapped charges at cavity walls (interfaces between internal defects, generally a cavity, and bulk insulation, where positive and negative charges are trapped as a result of electron avalanches, which constitute PD pulses) [6,7]. In the case of surface discharge, since the surface conductivity is, in general, significantly larger than the bulk one, faster de-trapping of charges is allowed, and, thus, the memory effect is partly lost and PD pulses occur when the voltage increases (in absolute value) and exceeds the PD inception field. 

From such premises, it is clear that without proper synchronization, PD pattern interpretation can fail. Interpretation or, better, identification, consists mainly in understanding the type of defect generating PD (internal, surface or corona), thus PD harmfulness and their contribution to accelerate (extrinsic) aging. It is straightforward that proper identification is the basis for a correct and effective condition-based maintenance plan.

Synchronization can be obtained by low frequency current transformer or Rogowski coil (synchronized with load current, thus the zero crossing must be corrected to refer to voltage/field), by quadrupoles connected to a capacitive sensor (separating the from high frequency components, the former connected to PD detector synchronization channel, the latter providing high-frequency noise or PD signals), as well as from electric field sensors.

However, in some industrial and transportation (e.g., ships) applications, where AC/DC or AC/AC converters are present for a significant share of the total power, voltage waveform can be affected by notches and harmonics (e.g., 5th and 7th). This can often be due to bridge switch commutation overlap (notches), large equivalent transformer impedance (thus harmonic current drop), and parallel resonance (harmonic voltage components amplification) [8,9,10]. Harmonic voltage components can accelerate intrinsic electrical degradation of electrical insulation systems [11,12] and cause an even faster breakdown if PD are incepted (extrinsic aging) [13,14].

The presence of notches and high-frequency harmonics in the voltage supply waveform can affect both synchronization, through failed zero-crossing detection, and identification. Mis-identification can occur due to both imprecise synchronization and the presence of a variable rate of change in voltage (dV/dt) in the voltage waveshape, which can change PD occurrence compared to AC sinusoidal voltage [15]. Both experts and possible statistic/AI analytics embedded in PD identification software can, therefore, fail to provide an adequate answer regarding PD presence and harmfulness. Work on the impact of voltage harmonic distortion is well summarized in [16], referring to total harmonic distortion (THD), which is a quantity related to global harmonic presence in the AC voltage waveform. The way harmonic voltage components can influence phase-resolved PD (PRPD) pattern and how this can impact PD measurement output, increasing THD, is illustrated in detail for AC voltage, with notes regarding DC. Actually, THD is a fundamental information to evaluate increased power losses, but only partially indicative of the risk of PD inception. In addition, the way harmonic voltages may affect PD patterns and make PD recognition more or less complex is also, and often prevailingly, related to the PDIV level compared with testing (operating) voltage. Simplifying, it can be speculated that when PD associated with harmonic voltages is just a part of the whole sub-PRPD pattern associated with PD, that is, most PD events occur due to the fundamental AC component of the voltage waveform, PD recognition can be handled by both an expert and an expert system quite straightforwardly. However, when most of the recorded PDs occur during dV/dt generated by harmonics and voltage notches, the PRPD pattern could hardly be recognized as due to PDs because its intrinsic characteristics are far from those expected under a sinusoidal waveform (e.g., phase shift, amplitude and phase dispersion, repetition rate). This is indeed the central contribution brought about by this paper, i.e., how to handle cases of PD due to voltage distortion, which could be problematic for experts or existing PD analytics software, developing automatic algorithms, AI-based, which would work in an unsupervised fashion, for PD-based condition monitoring.

This paper aims to provide an original contribution, focusing on PD sensing, phenomenology, and diagnostics under non-sinusoidal voltage waveforms, where harmonics and notches are present. PD measurements and analysis are performed on a cable with artificial defects, able to generate internal and surface discharges, and a motor, using a voltage waveform synthetically created using a function generator and an HV power amplifier. Two types of waveforms are considered here, that is, one with 5th and 7th harmonics and one with voltage notches, to highlight PD behavior when variation in dV/dt (voltage waveshape derivative) occurs slowly (harmonic) and abruptly (notches).

The major emphasis is on PD detection, analysis, and related identification. Due to harmonic distortion and voltage notches, PD can occur with phase and modality different from sinusoidal AC, even if the extent of harmonics and voltage notches is not large. This could affect the capability of human experts or AI software to understand the PD typology, that is, the type of defect generation PD. PD magnitude alone is somewhat useless if not associated with the knowledge of the type of defect from which they are generated, mostly whether it is internal (cavities embedded in insulation) or surface (e.g., PD generated at triple points). Indeed, the former is generally more harmful than the latter [6,17], thus diagnostics must account for it when developing condition maintenance plans. At the very end, the purpose of PD measurement/monitoring is to understand the risk of premature failure and the extent of accelerated aging, thus their harmfulness for insulation life. This should be the reason justifying investments in condition-based maintenance (CBM) relying upon PD assessment. As a general note, the results reported here are primarily qualitative, and no quantitative metrics (such as accuracy and precision) are considered. Comparison with human expert capabilities is, however, mentioned.

Section 2 will present the testing ground, Section 3 PD measurement results, associating dV/dt variation to PD occurrence, and Section 4 will discuss experimental results in the light of PD typology identification and relevant harmfulness. New software for PD detection and analysis will be presented for this purpose.

## 2. Test Set and Procedures

**Figure 1:**
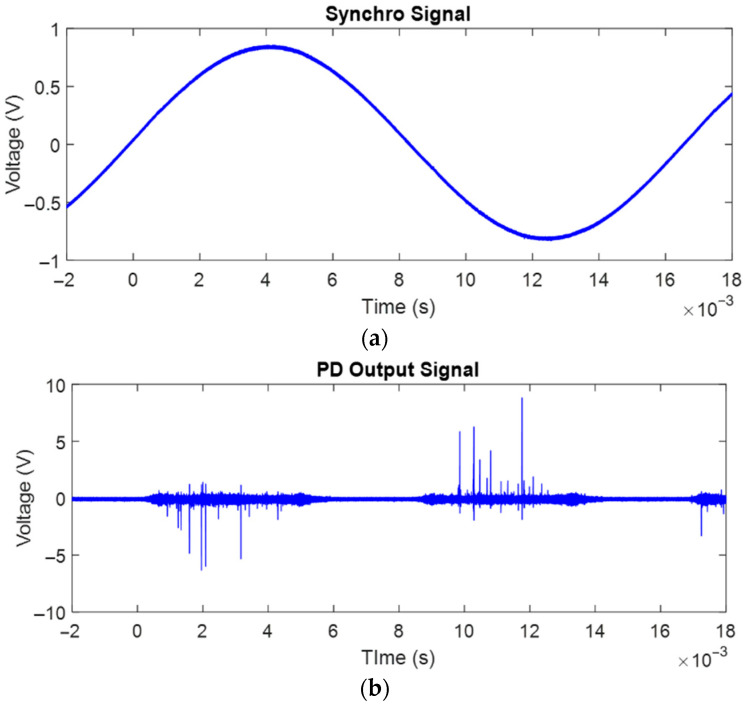
Quadrupole characteristic of the 2 nF coupling capacitor: (**a**) low-frequency (or synchro) output and (**b**) high-frequency (or PD) output.

The core components of the test set are the HV power amplifier, combined with a function generator, and an innovative PD system that is able to provide automatic recognition and identification of acquired PD signals. The power amplifier can be fed by almost any type of voltage waveform, from DC to AC sinusoidal, power electronics (as PWM supply), and AC non-sinusoidal, as in this paper. The output voltage is up to 30 kV, max dV/dt is 800 V/us, and maximum modulation frequency is 5 kHz. The function generator is programmed through MATLAB (version: R2022b; MathWorks, Natick, MA, USA). PD sensing is achieved through both an HFCT and a capacitive coupler (with two different capacitance values, i.e., 80 pF and 2 nF). In the first case, synchronization is provided by the power amplifier itself; in the second, a quadrupole was connected to the ground side of the capacitor. The quadrupole characteristic is shown in Figure 1a, where examples of signals coming from syncro and PD ports are also plotted. PD measurements are performed by a PD detector (HPM601; Rugged Monitoring, Quebec city, QC, Canada). with a bandwidth from 10 kHz to 100 MHz and a sampling rate of 250 MSa/s. It is driven by innovative software (version: 1.5.3-M; Seiktron, Weston, FL, USA) providing fully automatic acquisition and analytics (the latter according to the SRI (Separation, Recognition and Identification) approach [18,19]). The output is not only PD magnitude and repetition rate, but also effective noise rejection (through separation and recognition) and PD typology identification (internal, surface, corona in descending order of harmfulness). The latter relies upon AI (fuzzy logic) algorithms, so that identification is associated with the likelihood of PD source identification. It could be 1 (certain identification) or, for example, internal 80% and surface 20% when the AI system, emulating human expert thinking, sees the presence of both typologies in the denoised PD pattern [20].

**Figure 2:**
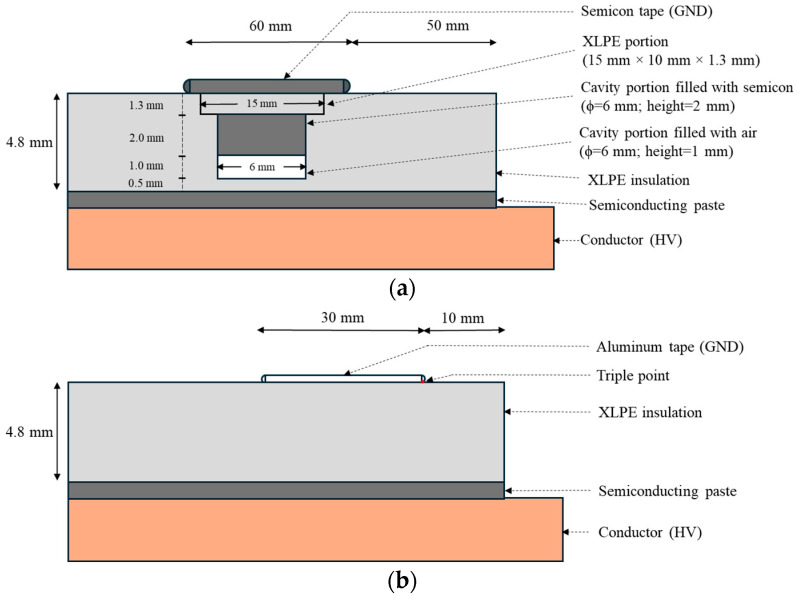
Sketch of (**a**) internal and (**b**) surface defects created on tested MV polymeric cable.

The test objects were an MV cable and an LV motor. The polymeric cable had two types of artificial defects, that is, internal and surface, as shown in Figure 2. The former was created by drilling a hole in cable insulation, partly filled by semicon (Figure 2a), the latter taping an aluminum foil (with borders folded suitably to control the curvature radius) on the cable insulation surface (Figure 2b). The motor had no artificial defects.

**Figure 3:**
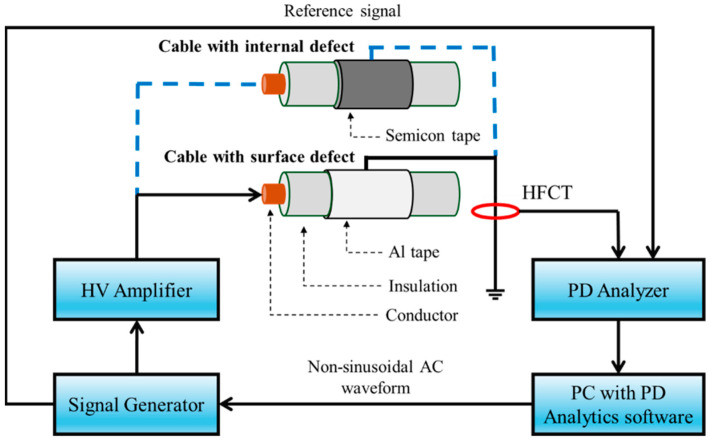
Scheme of the test system.

Figure 3 displays a scheme of the test system with a cable (where the two types of defects are highlighted) as a test object. PDs were sensed through an HFCT connected to the test object ground lead. PDIV was measured by increasing the applied voltage by 100 V till PD pulses were observed steadily (according to IEC 60270 [21]).

**Figure 4:**
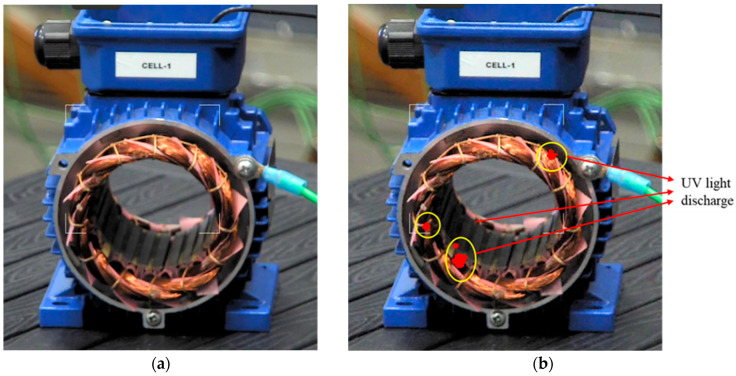
Example of UV light observation on motor (**a**) below and (**b**) above the PDIV. The red spots in (**b**) indicate UV emission (PD occurrence).

PD were measured and also observed by a ultraviolet (UV) camera, to associate the PD inception voltage (PDIV) with the appearance of light signals. Figure 4 shows an example of combined PDIV measurement results and UV signals, below and above PDIV. In general, with surface defects (where light is observable), PDIV and light onset coincide.

## 3. Results

**Figure 5:**
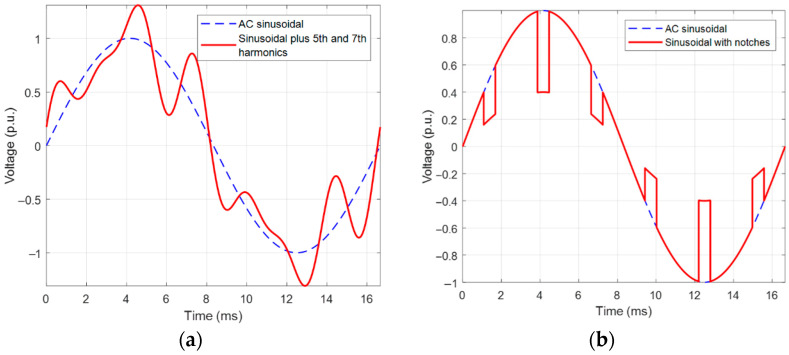
Types of supply voltage waveforms considered for PD investigation: sinusoidal plus (**a**) 5th and 7th harmonics (phase shifted by 60°), and (**b**) voltage notches.

Two types of waveforms were reproduced, with voltage harmonics and notches (Figure 5). The former consisted of overlapping 5th and 7th harmonic voltages to the AC sinusoidal, with amplitudes of 20% and 15%, respectively, of the maximum fundamental voltage and with variable phase shift. This would be the case of the voltage supply of an industrial plant where 6-pulse converters rectify the sinusoidal voltage (possibly followed by DC/AC converters) [8,9]. The other type of distortion caused by converters is voltage notches due to the overlap of switching on and off of bridge branch components [10], which was the second case considered for PD investigation.

**Figure 6:**
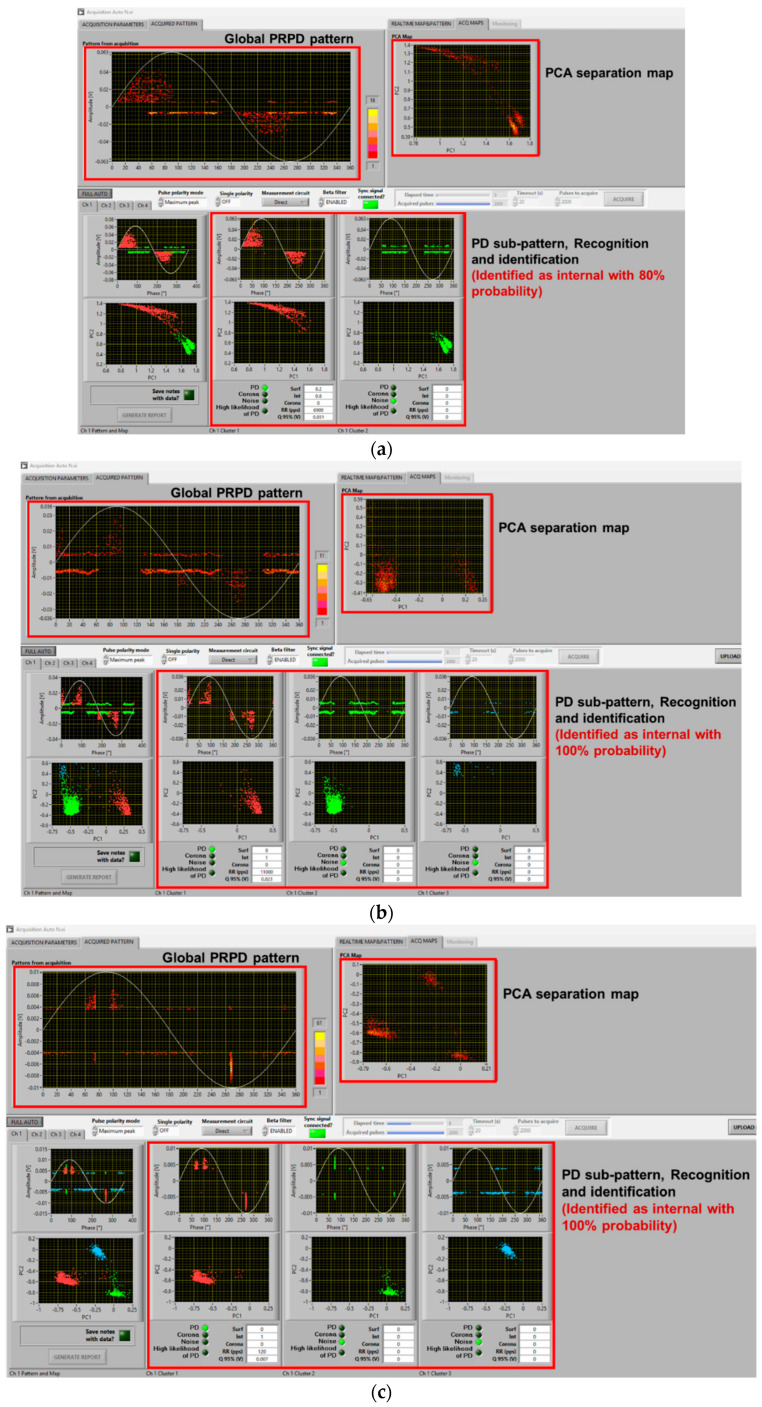
Example of software screenshots from (**a**) AC sinusoidal voltage, (**b**) sinusoidal voltage plus harmonics (Figure 5a), and (**c**) sinusoidal voltage with notches (Figure 5b) at 1.5 PDIV. Cable with internal defect.

**Figure 7:**
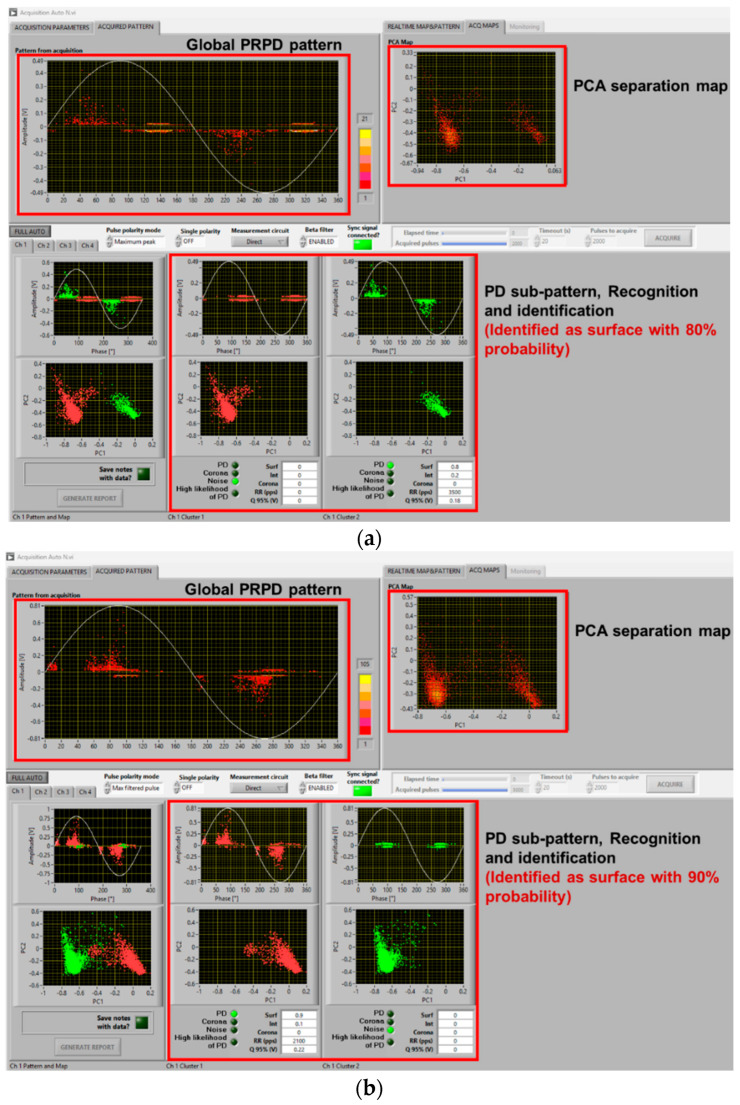

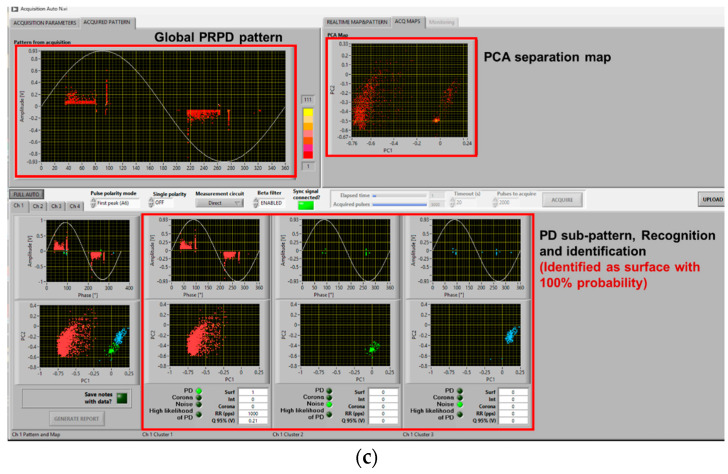
Example of software screenshots from (**a**) AC sinusoidal voltage, (**b**) sinusoidal voltage plus harmonics (Figure 5a), and (**c**) sinusoidal voltage with notches (Figure 5b), at 1.5 PDIV. Motor.

**Figure 8:**
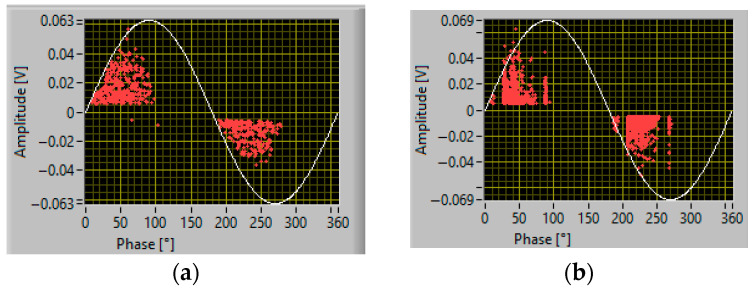
PD event in voltage half cycle, 1.5 PDIV: (**a**) AC sinusoidal voltage and (**b**) AC sinusoidal with notches (Figure 5b). Cable with internal defect. PD pulses are occurring often during voltage notches (dV/dt).

An example of the results of PD measurements with AC sinusoidal and the two types of waveforms described in Figure 5, for the cable with an internal defect and the motor, at 1.5 PDIV, is reported in Figure 6 and Figure 7, respectively. Major observations involve phase-resolved PD (PRPD) pattern shape and identification of PD typology. The PRPD pattern is a mix-up of a conventional AC sinusoidal pattern, which has a physics-based shape and comb-like PD events occurring when dV/dt varies due to harmonic voltages and/or notches. The latter phenomenon is similar to that occurring under PWM voltage supply, when PD pulses mostly occur during switching events, i.e., dV/dt steep increase or decrease [22,23,24]. An example of PD event distribution for a voltage waveform without (a) and with notches (b) is displayed in Figure 8, for the cable with an internal defect, 1.5 PDIV. As can be seen, PD events are mostly recorded during notch rise time, but some events are also present near zero crossing, for both AC sinusoidal and notched voltage (as expected for internal discharges). This behavior explains why the global pattern may account for conventional PD fingerprints, but also vertical bars (that is, PD events occurring at the same phase angle, with variable magnitude) can be present in correspondence with notches.

In such conditions, depending upon test voltage level (i.e., overvoltage compared to PDIV) and synchronization, identification of the type of source (*viz* internal surface, corona) generating PD may be complicated even for experts. This is discussed in the next section. Here, it is worthy noting that the innovative automatic acquisition and analytics software used for this investigation is robust and effective enough to provide, even in the presence of comb-like pattern structure, satisfactory identification of the PD source typology. Indeed, Figure 6 correctly identifies PD typology as mostly internal (likelihood 100%), while Figure 7 associates PD with surface discharges (likelihood 90%), as observed also through UV light emission.

## 4. Discussion

**Figure 9:**
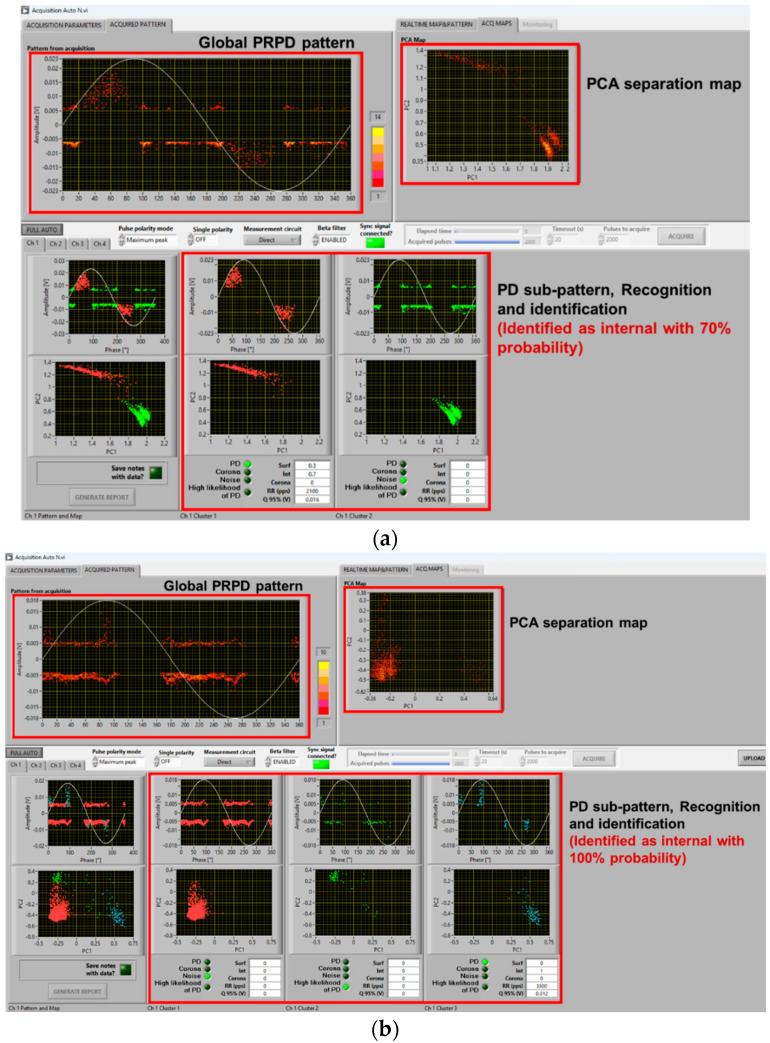

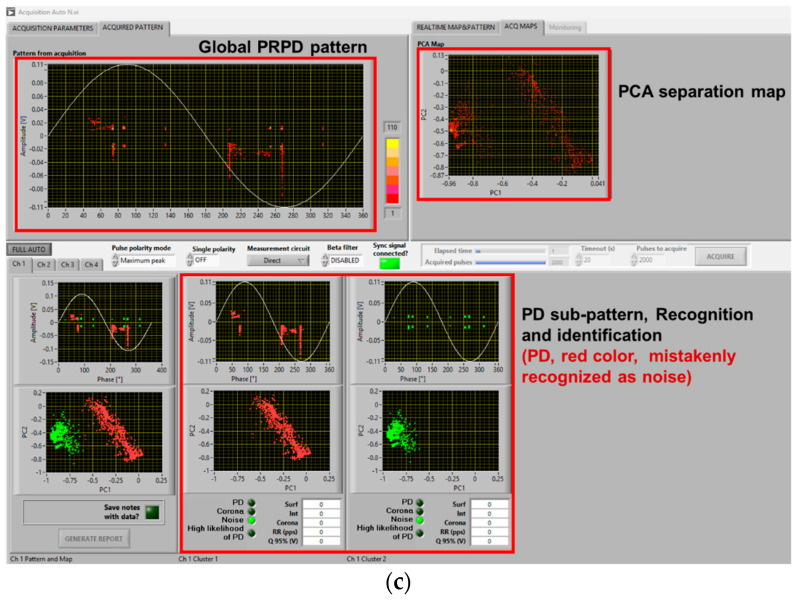
Example of software screenshots from (**a**) AC sinusoidal, (**b**) sinusoidal voltage plus harmonics (Figure 5a), and (**c**) sinusoidal voltage with notches (Figure 5b) at 1.1 PDIV. Cable with internal defect.

**Figure 10:**
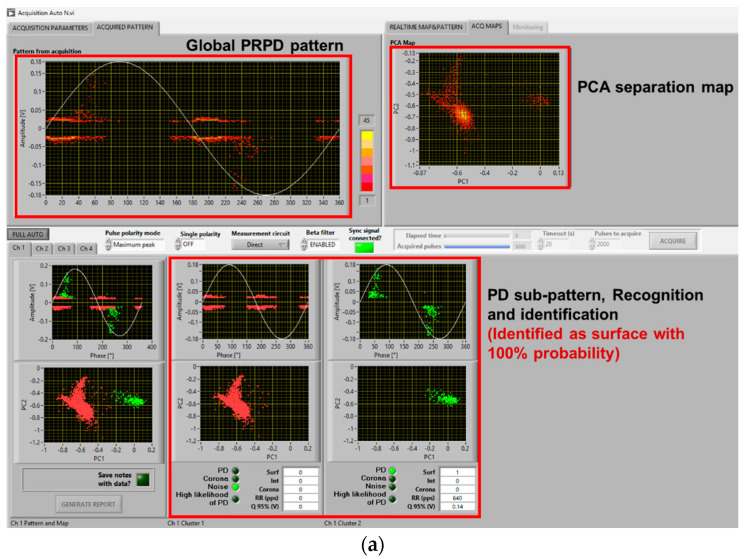

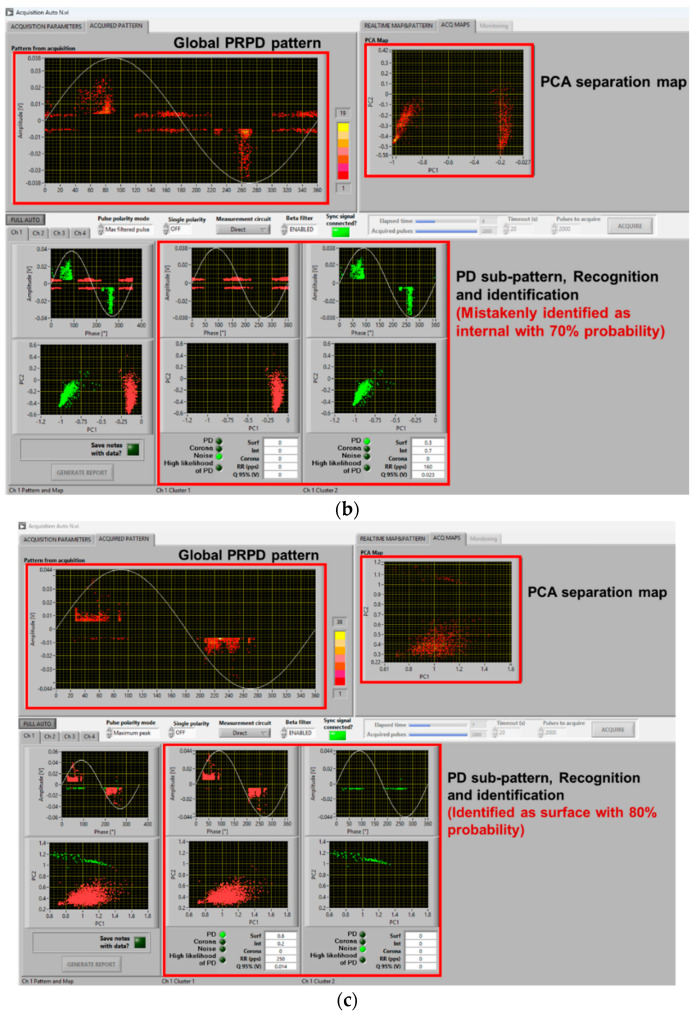
Example of software screenshots from (**a**) AC sinusoidal, (**b**) sinusoidal voltage plus harmonics (Figure 5a), and (**c**) sinusoidal voltage with notches (Figure 5b) at 1.1 PDIV. Motor.

As seen in the previous section, PD typology identification can be achieved successfully, at least by the developed fuzzy logic system used in acquisition and analytics software, with a distorted voltage supply. This likely holds even if synchronization with zero crossing is biased by a few degrees due to a voltage notch or harmonic component, and mostly, at 1.5 PDIV or, in general, at supply voltage values higher than PDIV. It can be speculated that close to PDIV partial discharge events may occur mostly during dV/dt variation, generating a kind of unconventional pattern that is mostly due to comb-like structures. This could jeopardize AI-based PD typology identification techniques and even human expert capability in tracking the type of source generating PD. As an example, Figure 9 and Figure 10 show the same cases of Figure 6 and Figure 7 but at 1.1 PDIV. The contribution to the PRPD pattern of the AC sinusoidal component vanishes, and mostly PD are incepted when dV/dt varies (increases) from the AC sinusoidal value. Hence, it becomes difficult to interpret the PRPD pattern, after denoising, for the sake of identification. The latter is, indeed, based on PD pattern features, such as pattern shift from zero-crossing voltage (e.g., PD occurrence ahead of zero and small amplitude dispersion are characteristics of internal discharges [7,11]).

**Figure 11:**
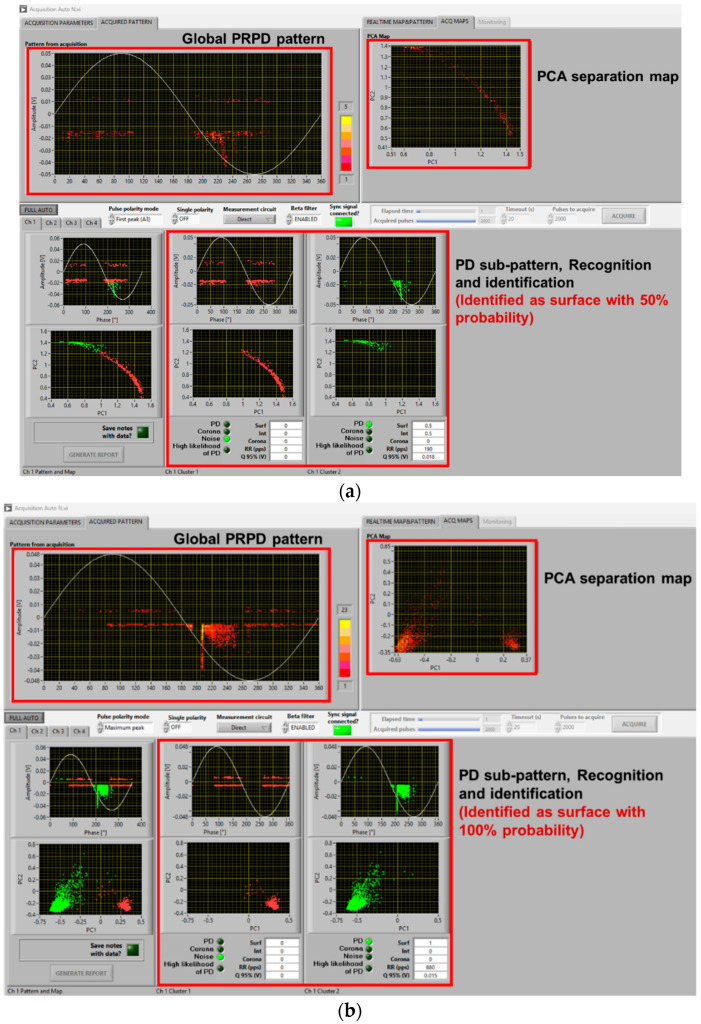
Automatic software screenshot for the case of cable with surface defect, at (**a**) 1.1 PDIV and (**b**) 1.5 PDIV for voltage notched waveform (Figure 5b).

Comparing Figure 9a with Figure 9b,c and Figure 10a with Figure 10b,c, it is emphasized that in some cases identification may fail, misinterpreting PD as noise/disturbance. In practice, the more the PRPD pattern differs from the conventional one, known under AC sinusoidal voltage and constituting the basis for expert evaluation, the more challenging PD analysis and harmfulness assessment. This is the message conveyed by Figure 9, Figure 10 and Figure 11, highlighting that at 1.1 PDIV the PRPD pattern might not be anymore related to the fundamental voltage zero crossing, so that it could, sometimes, be wrongly associated with noise or disturbance (that is, signals not related to PD or generated by PD, but outside the object under test).

**Figure 12:**
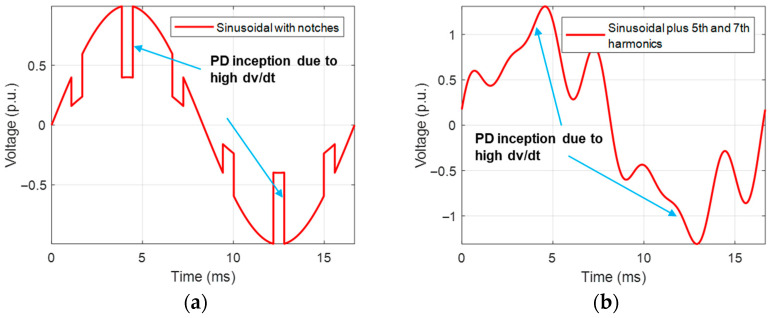
Conditions for PD inception during dV/dt variation due to (**a**) voltage harmonic components and (**b**) voltage notches.

The type of PD pattern that can be induced by voltage harmonics and notches resembles the characteristic (comb-like pattern) of the PWM waveform, analyzed in [15]. When dV/dt varies steeply from the AC sinusoidal behavior, the electrical field on surface defects or internal cavity has a fast variation, which may cause PD inception (compatibly with the availability of the firing electric, the major stochastic component to PD occurrence). This is summarized in Figure 12a, relevant to 5th and 7th voltage harmonics overlapped to the fundamental (Figure 5) and to voltage notches, Figure 12b. Under AC sinusoidal voltage affected by notches, and for the same magnitude of peak voltage, an increase in notch depth leads to higher voltage variation during the transient (jump voltage during the notch rise/fall time). As the jump voltage exceeds a critical threshold value, PD can be initiated during most transients (due to the notches), resulting in a sudden rise in the repetition rate [24].

The challenge is, therefore, to modify the analytics software, which performs properly under sinusoidal AC and when the nominal voltage is well higher than PDIV, for those cases where the nominal voltage is close to PDIV and PD signals can be confused by noise/disturbance (by both AI and the human experts). Separation and Recognition would work well, being based on quantities associated with the acquired signals, thus analyzing each pulse. Identification, which uses PRPD de-noised sub-pattern and is based on AI (fuzzy logic), may be affected, as shown in Figure 9c and Figure 10b.

**Figure 13:**
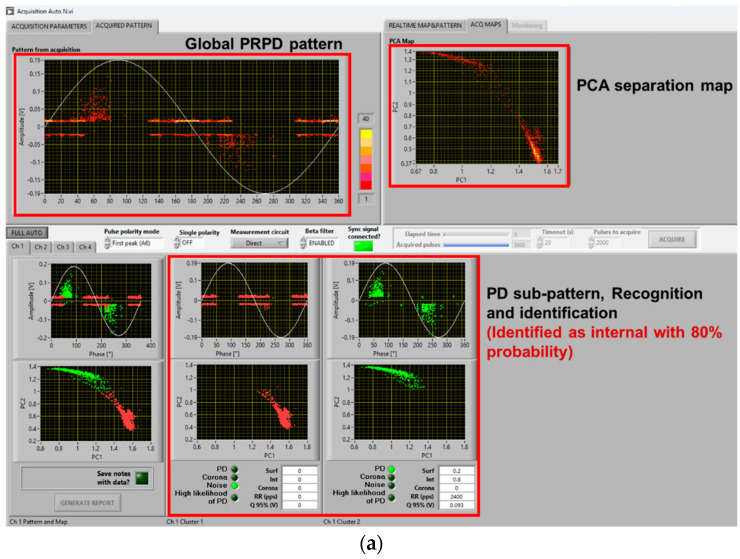

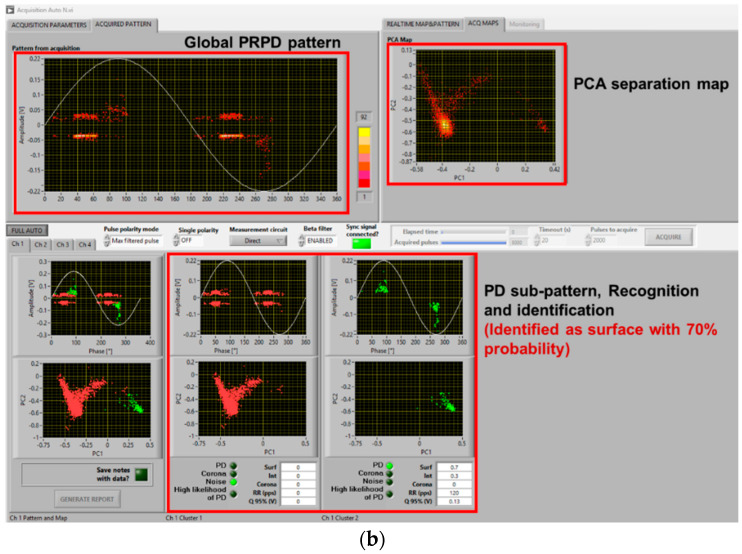
Automatic software screenshot with proper identification for the cases of (**a**) Figure 9c and (**b**) Figure 10b, with the modified software.

A solution is to modify some of the fuzzy rules, especially those related to pattern phase shift. Indeed, if PDs mostly occur during dV/dt variation, PD events can be far from voltage zero crossing even if they are generated by internal defects (cavities). The opposite can occur for surface discharges. Also, PD magnitude dispersion (standard deviation or shape parameter of the Weibull function [20]) can be affected by dV/dt, as shown by the contribution of comb-like structures in the PRPD pattern with voltage harmonics and notches. This can be addressed by modifying the level of logic fuzziness, that is, reducing the slope of membership interactions. An example of amended software behavior, related to the results of Figure 9c and Figure 10b, is shown in Figure 13. As can be seen in Figure 13a, PD pulses are recognized and identified as mostly internal with a likelihood of 80%, while in Figure 9c, it was mistakenly recognized as noise. Similarly, in Figure 13b, PD pulses are recognized and identified as mostly surface with 70% likelihood, while it was mistaken as internal with 70% likelihood in Figure 10b.

**Figure 14:**
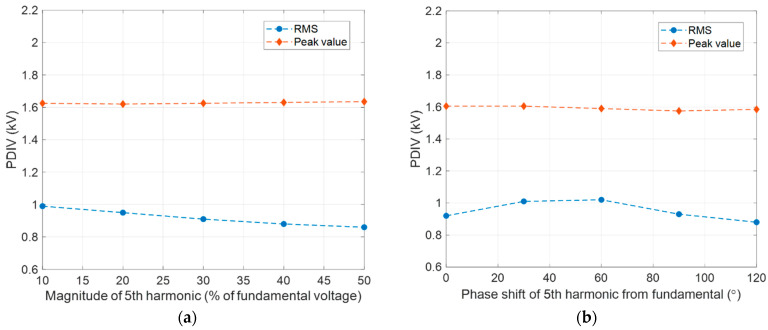

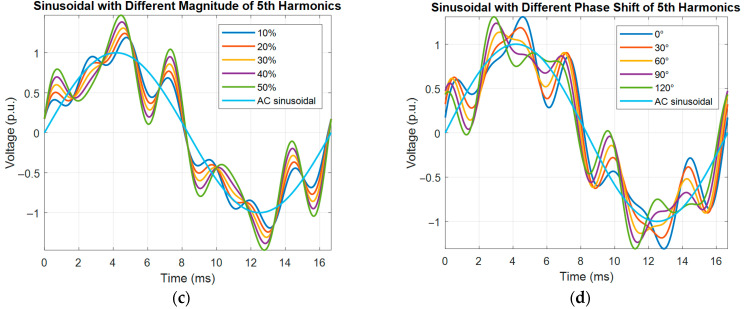
Behavior of PDIV under AC sinusoidal supply and with the presence of harmonic voltage components (5th and 7th), where the 5th harmonic (**a**) increases in magnitude and (**b**) shifts from the fundamental (60 Hz); corresponding waveforms of applied supply voltage with harmonic, where the 5th harmonic varies in (**c**) magnitude and (**d**) shifts from the fundamental (60 Hz). The RMS and peak value of input voltage waveform corresponding to the PDIV are reported.

**Figure 15:**
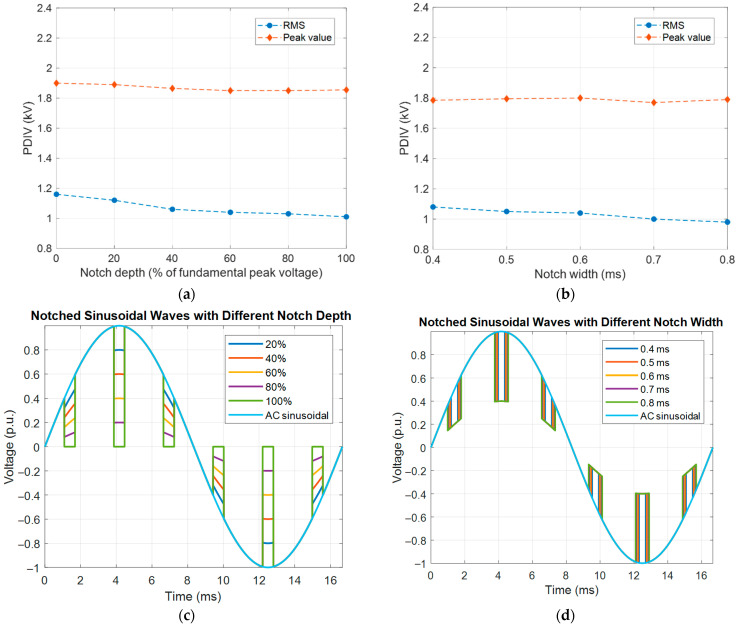
Behavior of PDIV under AC sinusoidal supply and with notches, with varying (**a**) notch depth and (**b**) notch width; corresponding waveform of applied supply voltage with notches, varying notch (**c**) depth and (**d**) width. The RMS and peak value of input voltage waveform corresponding to the PDIV are reported.

A last observation is devoted to the possible modification of PDIV due to voltage distortion. Figure 14 shows the behavior of PDIV comparing AC sinusoidal supply with the case of the presence of harmonic voltage components (5th and 7th), where the 5th harmonic increases in magnitude and shifts from the fundamental (60 Hz) zero voltage. Similarly, Figure 15 displays the PDIV variation with depth and width of voltage notches. Each figure (Figure 14 and Figure 15) reports the RMS and peak value of input voltage waveform corresponding to the PDIV. As can be seen, while the peak value has little change with harmonic content in magnitude and phase, the RMS value can decrease noticeably, thus it might be unpredictable in real conditions.

## 5. Conclusions

Voltage distortion is not just associated with increased power losses (due to harmonic currents), but it can also, and in particular, cause potential accelerated intrinsic and extrinsic aging. Losses are, indeed, related to the RMS electrical field (which can exceed that under AC sinusoidal depending on the phase shift in voltage harmonics and notches), aging acceleration to increased maximum voltage (intrinsic aging), and partial discharge phenomena (extrinsic aging).

Focusing on PD, this paper shows how much their occurrence is modified by the type and extent of voltage distortion, which can generate significant issues in PD measurement interpretation (from both experts and AI systems) and relevant maintenance action planning. Sensing and synchronization are significant factors, but developing, as shown here, a smart analysis offering the identification of the type of defect generating PD, as well as providing an enhanced noise rejection feature, is a must.

First, it is seen that PD can be mistaken for noise (or, sometimes, and with some types of commercial detectors, the opposite). Then the identification of the type of source generating PD, which is fundamental to establish PD harmfulness (thus again to plan properly maintenance action), can be wrong or clueless. Eventually, PDIV can vary with the extent and type of distortion, compared to the AC sinusoidal, even if the AC fundamental voltage component does not change.

As a positive note, it is shown that the automatic analytics software developed and used in this paper, suitably modified from its AC sinusoidal version, becomes effective for PD identification even in cases where the unconventional pattern generated by distortion (notch, harmonics)-related PD is largely predominant in the whole PRPD pattern. This, however, is the takeover of the artificial waveforms created in the lab to carry out this research. In practice, different harmonic waveforms can be present, thus results reported here must be taken with care (even if waveforms as those reported in Figure 5 were derived from real on-field voltage measurements).

A general warning is that the presence of voltage distortion components can reduce PDIV, increase PD magnitude and repetition rate, and, therefore, the risk of extrinsic accelerated aging in electrical or electronics components with respect to AC sinusoidal conditions. Hence, insulation system design must account for it for the sake of reaching the reliability and life specifications for electrical asset components.

Final note: Only the analysis of each single detected pulse could help an expert to understand if PDs are present, but this cannot be clearly carried out by an unsupervised monitoring system. This paper has the merit of showing the feasibility of an automatic approach, fundamental for condition monitoring, which can get rid of expert evaluation needs at each PD acquisition.

## Data Availability

The original contributions presented in the study are included in the article; further inquiries can be directed to the corresponding author.

## Abbreviations

The following abbreviations are used in this manuscript:

CBM	Condition-Based Maintenance
HFCT	High-Frequency Current Transformer
PD	Partial Discharge
PDIV	Partial Discharge Inception Voltage
PRPD	Phase-Resolved Partial Discharge
PWM	Pulse Width Modulation
SRI	Separation, Recognition and Identification
UV	Ultraviolet

## References

[1] (2011). Evaluation and Qualification of Electrical Insulation Systems.

[2] Stone G.C. (2005). Partial discharge diagnostics and electrical equipment insulation condition assessment. IEEE Trans. Dielectr. Electr. Insul..

[3] Dissado L.A., Fothergill J.C. (1992). Electrical Degradation and Breakdown in Polymer.

[4] Bartnikas R. (2002). Partial discharges. Their mechanism, detection and measurement. IEEE Trans. Dielectr. Electr. Insul..

[5] Krivda A. (1995). Automated recognition of partial discharges. IEEE Trans. Dielectr. Electr. Insul..

[6] Kreuger F.H. (1990). Partial Discharge Detection in High-Voltage Equipment.

[7] Pattanadech N., Haller R., Kornhuber S., Muhr M. (2023). Partial Discharges (PD): Detection, Identification and Localization.

[8] Kimbark E.W. (1971). Direct Current Transmission.

[9] Arillaga J., Watson N.R. (2003). Power System Harmonics.

[10] Montanari G.C., Loggini M. (1987). Voltage-distortion compensation in electrical plants supplying static power converters. IEEE Trans. Ind. Appl..

[11] Cavallini A., Ghinello I., Mazzanti G., Montanari G.C. (2002). Considerations on the life performance and installation practice of shunt capacitors in the presence of harmonics generated by AC/DC converters. IEEE Trans. Power Deliv..

[12] Fabiani D., Montanari G.C. (2002). The effect of voltage distortion on ageing acceleration of insulation systems under partial discharge activity. IEEE Electr. Insul. Mag..

[13] Stone G.C., Culbert I., Boulter E.A., Dhirani H. (2014). Electrical Insulation for Rotating Machines: Design, Evaluation, Aging, Testing, and Repair.

[14] Montanari G.C., Hebner R., Morshuis P., Seri P. (2019). An approach to insulation condition monitoring and life assessment in emerging electrical environments. IEEE Trans. Power Deliv..

[15] Jiang T., Cavallini A., Montanari G.C., Li J. Analysis of partial discharge activity in line-commutated HVDC converter. Proceedings of the International Conference on High Voltage Engineering and Application.

[16] Florkowski M. (2022). Influence of Harmonics on partial discharge measurements and interpretation of phase-resolved patterns. Measurement.

[17] (2021). Rotating Electrical Machines—Part 27-5: Off-Line Measurement of Partial Discharge Inception Voltage on Winding Insulation Under Repetitive Impulse Voltage.

[18] Seri P., Ghosh R., Montanari G.C. (2021). An unsupervised approach to partial discharge monitoring in rotating machines: Detection to diagnosis with reduced need of expert support. IEEE Trans. Energy Convers..

[19] Myneni S.B., Montanari G.C. (2024). Estimation of the Partial Discharge Inception Voltage of Electrical Asset Components at Variable Environmental Pressure: A Modelling Approach. Energies.

[20] Contin A., Cavallini A., Montanari G.C., Pasini G., Puletti F. (2002). Digital detection and fuzzy classification of partial discharge signals. IEEE Trans. Dielectr. Electr. Insul..

[21] (2015). High-Voltage Test Techniques—Partial Discharge Measurements.

[22] (2014). Partial Discharge Free Electrical Insulation Systems (Type I) Used in Rotating Electrical Machines Fed from Voltage Converters—Qualification and Quality Control Tests.

[23] Kimura K. The role of IEC 60034-27-5 for IEC 60034-18-41: Offline PD test methods with repetitive impulse voltage. Proceedings of the International Symposium on Electrical Insulating Materials (ISEIM).

[24] Seri P., Montanari G.C. (2019). A voltage threshold in operating condition of PWM inverters and its impact on reliability of insulation systems in electrified transport applications. IEEE Trans. Transp. Electrif..

